# Exploring Augmented Reality for Dental Implant Surgery: Feasibility of Using Smartphones as Navigation Tools

**DOI:** 10.1002/cre2.70110

**Published:** 2025-03-05

**Authors:** Richard Mosch, Vasilios Alevizakos, Dragan Alexander Ströbele, Marcus Schiller, Constantin von See

**Affiliations:** ^1^ Department of Dentistry, Faculty of Medicine and Dentistry, Research Center for Digital Technologies in Dentistry and CAD/CAM Danube Private University Krems an der Donau Austria; ^2^ Department of Oral and Maxillofacial Surgery Hannover Medical School Hannover Germany

**Keywords:** augmented reality, dental implants, image‐guided surgery, smartphone

## Abstract

**Objectives:**

Dental implant placement requires exceptional precision to ensure functional and esthetic success. Traditional guidance methods, such as static drilling guides and dynamic navigation systems, have improved accuracy but are limited by high costs, rigidity, and reliance on specialized hardware. This study introduces an augmented reality (AR) system using consumer smartphones for real‐time navigation in dental implant placement. The system aims to provide a cost‐effective, eco‐friendly alternative to conventional methods by integrating virtual planning with physical models.

**Material and Methods:**

A modified dental training model with removable parallel pins served as the physical component. Implant positions were digitally planned and color‐coded using 3D scanning and modeling software, then integrated into an AR application built with Unity Engine. A smartphone's camera was calibrated to project virtual overlays onto the physical model. In vitro testing evaluated alignment accuracy, drill guidance, and system performance under controlled lighting conditions.

**Results:**

The AR system successfully aligned virtual overlays with the physical model, providing effective visual guidance for implant drill positioning. Operators maintained planned trajectories, demonstrating the feasibility of AR as an alternative to static and dynamic guidance systems. Challenges included the system's sensitivity to stable lighting and visual cues.

**Conclusions:**

This AR‐based approach offers an accessible and sustainable solution for modern dental implantology. Future research will focus on quantitative accuracy assessments, AI integration for enhanced performance, and clinical trials to validate real‐world applicability. AR technology has the potential to transform dental practices by improving outcomes while reducing costs and environmental impact.

## 1 Introduction

The demand for dental implants has increased significantly in recent years, driven by patients seeking fixed restorations that enhance functionality, esthetics, and overall quality of life (Duong et al. 2022). Successful dental implant placement relies on precision, as the positioning of implants directly impacts both functional and esthetic outcomes (Nulty 2024). Advanced techniques, such as backward planning and computer‐guided surgery, have substantially improved accuracy and reduced failure rates while also streamlining workflows for clinicians and dental technicians (Kim et al. 2024). These approaches have become central to modern implantology, demonstrating significant advantages over earlier methods.

Currently, backward planning involves combining cone‐beam computed tomography (CBCT) data with virtual prosthetic designs to create custom surgical guides. These guides ensure reliable accuracy, with deviations typically limited to less than ±1 mm (Hama and Mahmood 2023). However, despite their effectiveness, traditional surgical guides are not without limitations. They lack the flexibility to adapt to unexpected intraoperative changes, and their production is both costly and wasteful, contributing to medical waste generation (Campos et al. 2024; Vu et al. 2024). Additionally, the physical presence of the guides can obstruct the clinician's view during surgery, which can complicate the procedure.

Dynamic navigation systems have emerged as a more flexible alternative, providing real‐time guidance without the need for physical templates (Younis et al. 2024). These systems, however, come with their own challenges, including reliance on expensive specialized equipment, such as optical trackers and dedicated workstations, which limits their accessibility in routine clinical practice. These challenges underscore the need for an innovative solution that balances precision, affordability, and ease of use.

Augmented reality (AR) represents a promising technology that could address these limitations (Puleio et al. 2024). By overlaying virtual models onto the real‐world surgical field in real time, AR can provide dynamic guidance without the need for physical surgical guides or costly specialized hardware (Jiang et al. 2018; Navab et al. 2022). Recent research has underscored AR's potential in precision tasks. For example, Dastan, Fiorentino, and Uva (2024) reviewed tools like the Precise Tool to Target Positioning Widgets (TOTTA), which demonstrated AR's capability to enhance spatial accuracy in surgical workflows. Another study by Dastan, Fiorentino, Walter (2024) explored the codesign of mixed reality drill positioning systems with dentists, emphasizing AR's ability to function effectively in realistic clinical setups. These findings establish AR as a transformative technology for medical and dental procedures. Additionally, AR has the potential to enhance accessibility by leveraging widely available consumer devices, such as smartphones (Lee et al. 2024). Despite its transformative potential, the application of AR in dentistry—particularly for dental implant placement—remains relatively unexplored, highlighting a significant gap in the current literature.

This study introduces a novel approach that combines AR‐based navigation with the use of smartphones to guide dental implant placement. By integrating virtual planning with real‐time augmented visualization, the proposed system aims to simplify implant procedures, reduce costs, and enhance accessibility. The research demonstrates the feasibility of this method through in vitro testing with a dental training phantom while also identifying key practical challenges, such as lighting conditions and optical obstructions, that could impact the system's performance. Furthermore, it explores the potential of smartphones as an affordable and environmentally sustainable alternative to traditional navigation systems.

## 2 Materials and Methods

This study evaluated the feasibility of using AR for guided dental implant placement by integrating virtual planning with physical modeling. The methodology included detailed steps for virtual implant planning, physical model preparation, AR application development, and in vitro testing under simulated clinical conditions. The following sections describe the materials and methods employed, ensuring reproducibility of the proposed approach.

### 2.1 Materials

#### 2.1.1 Dental Training Phantom Head

A dental training phantom head (PK‐1 TL, Frasaco GmbH, Tettnang, Germany) was used to replicate the anatomical and spatial constraints of the human oral cavity. This model is widely utilized for dental training and testing, featuring realistic representations of the jaw, gingiva, and teeth. Its stable design enabled controlled simulation of clinical conditions for evaluating the AR system.

#### 2.1.2 Dental Training Model

The physical representation for testing consisted of a dental training model (ANA‐4V, Frasaco GmbH, Tettnang, Germany). This model features full dentition and was specifically modified for the study by decapitating selected teeth to allow the placement of removable parallel pins at predetermined implant positions. The pins simulated the intended implant locations and served as references for aligning virtual and physical components.

#### 2.1.3 3D Scanning and Software Tools

The modified dental model was scanned using a high‐resolution 3D scanner (D800, 3Shape, Copenhagen, Denmark) to generate an accurate digital representation of the physical model. The resulting STL file was processed in Blender 2.9 (Blender Foundation, Amsterdam, Netherlands), where the virtual model was prepared for AR integration. This involved segmenting teeth, soft tissue, and pins, followed by color‐coding each component to facilitate identification during AR projection. The final file was exported in FBX format.

#### 2.1.4 AR Hardware and Application

A commercially available smartphone (Samsung Galaxy S10, Samsung Electronics Co. Ltd., Seoul, South Korea) equipped with an autofocus camera served as the AR platform. The AR application utilized the phone's built‐in object detection algorithms to recognize visual cues on the physical model and superimpose the virtual implant positions. This approach minimized the need for specialized hardware, making the system cost‐effective and accessible.

#### 2.1.5 Lighting Setup

Two LED lights (Andoer MS‐30L, Shenzhen Tomtop Technology Co. Ltd., Shenzhen, China) were used to ensure consistent and uniform illumination. Proper lighting was critical to enhance the visibility of the physical model and to prevent shadows or reflections that could interfere with the AR system's accuracy.

### 2.2 Methods

#### 2.2.1 Virtual Planning of Implant Positions

The first phase of the study involved virtual planning, in which optimal implant positions were determined based on the digital design of the prosthetic crown. Using Blender 2.9, the scanned STL file of the dental model was prepared by aligning three implant positions with the removable pins. These positions were digitally marked and color‐coded to differentiate them from surrounding anatomical structures. This meticulous planning ensured that the virtual model precisely corresponded to the physical setup, facilitating accurate AR projection.

#### 2.2.2 Preparation of the Physical Model

The physical model was modified by decapitating the crown portion of the tooth at position 26 (FDI system) and inserting three removable parallel pins. These pins simulated the planned implant positions, allowing for easy removal and replacement during testing. The modified model was used both for 3D scanning and for in vitro testing to ensure alignment between the virtual and physical domains.

#### 2.2.3 AR Setup and Calibration

**Figure 1:**
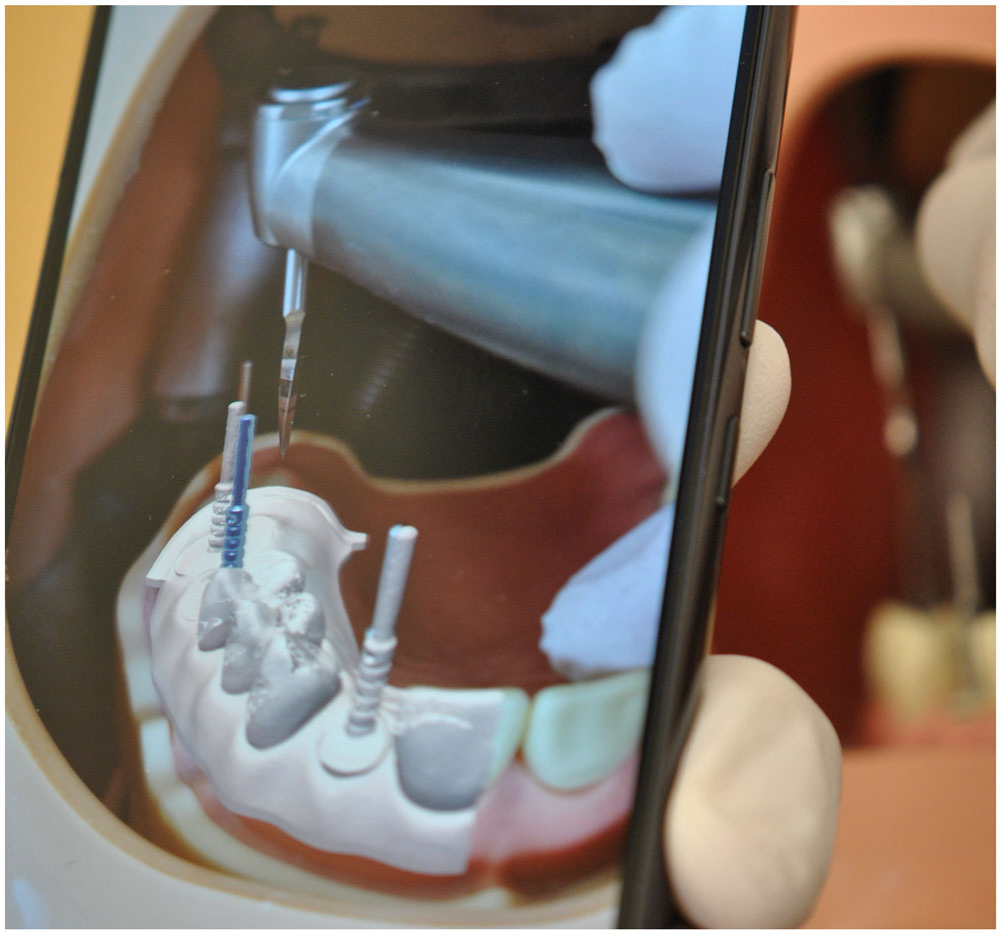
Superimposed model with virtually projected parallel pin onto the physical pin.

The AR application was designed to project virtual overlays of the implant positions onto the physical model using the smartphone's camera. Calibration was achieved by aligning the camera's view with the occlusal plane of the dental model, ensuring proper superimposition of digital and physical elements. The application relied on the color‐coded cues from the model to recognize key anatomical features and project the planned implant positions in the correct location and orientation (Figure 1).

#### 2.2.4 In Vitro Testing Protocol

**Figure 2:**
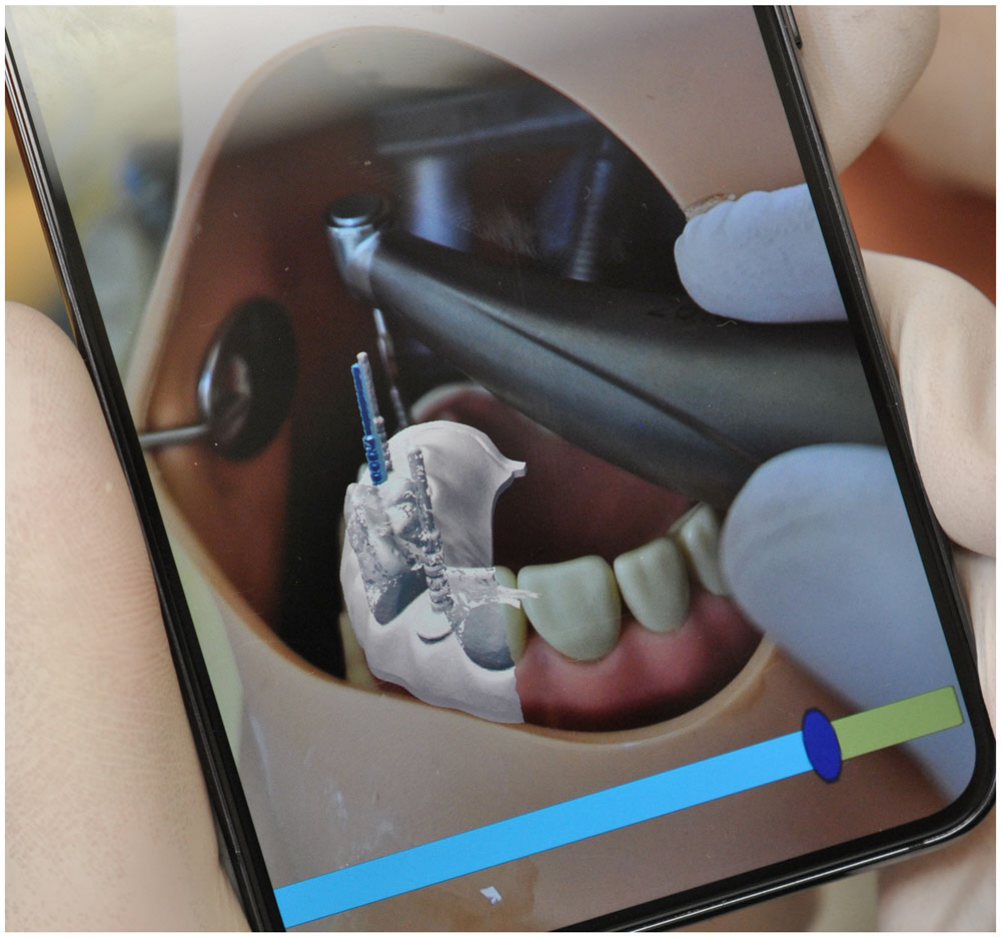
The application detected the pre‐planned digital implant placement.

**Figure 3:**
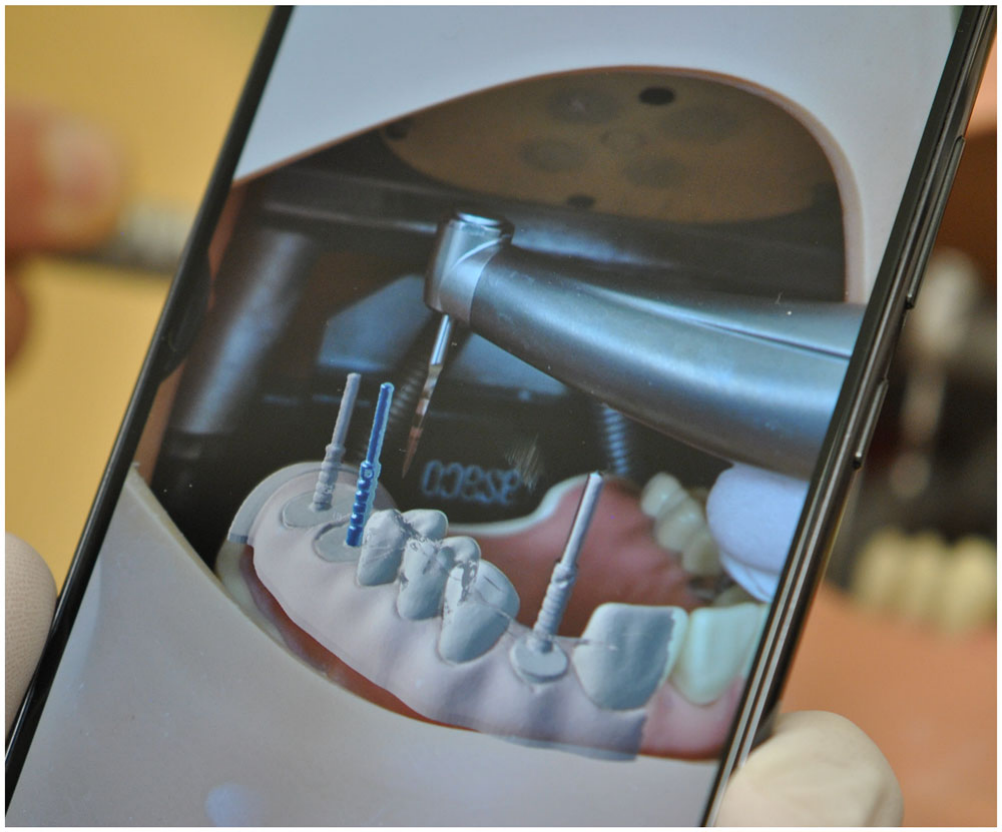
The AR application utilized the built‐in camera of a standard smartphone with autofocus to generate a mobile AR environment.

To evaluate the feasibility of the AR guidance system, the prepared dental model was placed within the phantom head, replicating the constraints of a clinical scenario. The protocol for testing included (Figures 2 and 3):

- Positioning the smartphone's camera perpendicular to the occlusal surface to optimize recognition of visual cues.
- Activating the AR application, which superimposed the pre‐planned digital implant positions onto the physical model.
- Removing the parallel pin at position 26 to simulate a missing implant site.
- Using the AR overlay to guide the implant drill into the correct position and angulation, as displayed on the smartphone screen.

Throughout the testing process, consistent lighting conditions were maintained to prevent errors in AR projection. Observations focused on the accuracy of alignment between the digital overlays and the physical model, as well as the usability of the system for guiding the implant drill.

## 3 Results

The feasibility of using AR for guided dental implant placement was evaluated through controlled in vitro testing. The experiments assessed the system's ability to align virtual implant positions with physical models, provide drill guidance, and maintain performance under varying conditions.

The AR system consistently projected virtual implant positions onto the physical model. The alignment of the virtual overlays with the predefined implant sites was visually verified during each trial.

Operators were able to visually maintain the position and angulation throughout the procedure, demonstrating the AR system's potential as a reliable alternative to traditional drilling guides.

## 4 Discussion

This study presents an innovative method for integrating mobile AR systems into dental implant placement, leveraging the accessibility and computational power of consumer smartphones. By utilizing AR to project virtual overlays onto physical models, this approach addresses key limitations of existing guidance methods, providing a cost‐effective, eco‐friendly, and adaptable alternative for clinicians.

### 4.1 Innovative Aspects of the Proposed Method

AR was chosen for its ability to dynamically overlay digital models onto physical structures in real time, a feature that overcomes the rigidity of traditional static drilling guides. (Wang et al. 2023). Dynamic navigation systems, although more versatile, require complex and expensive optical tracking equipment, which restricts their widespread adoption (Tang et al. 2024).

The AR system proposed in this study eliminates these barriers by leveraging the affordability and widespread availability of smartphones. The system requires no specialized hardware or extensive calibration, relying instead on consumer devices equipped with integrated cameras and computational capabilities. By integrating visual cues directly into the physical model, the AR application ensures precise alignment between virtual overlays and physical components. This real‐time visualization might enable clinicians to adjust implant positioning dynamically, improving procedural flexibility while reducing costs and environmental impact.

### 4.2 Feasibility Demonstrated in In Vitro Testing

The in vitro testing validated the system's feasibility, demonstrating consistent alignment of virtual overlays with physical models and effective guidance for implant drill positioning. The AR system promises maintained overlay clarity under proper lighting conditions, facilitating precise visualization of planned implant trajectories. These findings highlight the potential of AR as a cost‐effective and adaptable solution that combines the strengths of traditional and dynamic guidance systems without their associated limitations.

### 4.3 Camera Visibility and Target Recognition

Optimal visibility for the camera was achieved by calibrating the smartphone's autofocus to focus on the occlusal surface of the physical model. Predefined, color‐coded regions on the model served as visual targets, which the AR application recognized and mapped to corresponding digital overlays. This integration of visual cues ensured consistent alignment between the physical and virtual components, minimizing the risk of misalignment due to variations in camera angle or distance (Goutcher et al. 2022).

While the current system utilized computer vision algorithms to recognize and project overlays, future enhancements could incorporate AI‐based techniques to improve target recognition (Panayides et al. 2020). For instance, machine learning algorithms could enable the system to adapt to variable lighting conditions, occlusions from instruments, or operator movement, further enhancing the robustness of the AR system.

### 4.4 Challenges and Limitations

Despite its promise, the AR system faces certain challenges that must be addressed for successful clinical application. The reliance on stable lighting and unobstructed visual cues introduces potential vulnerabilities in dynamic surgical environments (Tsai et al. 2023). Shadows cast by instruments or uneven illumination occasionally disrupted the system's functionality during testing (Jakab et al. 2024). Future iterations could incorporate adaptive algorithms or improved lighting setups to mitigate these issues.

Another limitation lies in the operator‐dependent nature of the AR system. The smartphone's orientation significantly influenced the initial alignment of the virtual and physical models. A vertical orientation targeting the largest occlusal surface produced the most accurate results, while deviations from this position led to alignment errors (Cunningham and Brooks 2022). Standardized protocols or automated alignment features could reduce operator variability, ensuring greater consistency in clinical settings.

### 4.5 Eco‐Friendly and Economic Advantages

The proposed method aligns with sustainable practices by eliminating the need for disposable drilling guides, which generate medical waste and incur significant fabrication costs. By replacing physical templates with virtual overlays, the system reduces environmental impact while streamlining the implant placement workflow (Hölken et al. 2022). Furthermore, the reliance on consumer smartphones eliminates the expense of specialized hardware, making the system accessible to a broader range of dental practitioners. These features underscore the economic and ecological advantages of the AR approach, making it a viable option for modern dental practices.

### 4.6 Future Directions

This feasibility study was designed to explore the potential of an AR system for guiding dental implant placement, rather than to quantitatively assess its accuracy or compare it directly with existing methods. The focus of this initial phase was to demonstrate the basic functionality of the system, including the alignment of virtual overlays with the physical model and its ability to guide the implant drill visually.

Future research will include detailed measurements of accuracy, such as deviations between planned and achieved implant positions, as well as statistical analyses to validate the system's performance. Comparisons with current state‐of‐the‐art guidance systems will also be conducted to establish its clinical relevance. Additionally, AI‐based enhancements for automated target recognition, real‐time adjustments, and robustness against environmental variability will be integrated into the system to address current limitations.

Clinical trials will be essential to assess the system's effectiveness and usability in real‐world scenarios. Investigations into advanced hardware, such as depth sensors or stereo cameras, may further optimize the AR system for broader applications, ensuring reliable performance in diverse surgical environments.

## 5 Conclusion

This study demonstrates the feasibility of using AR on consumer smartphones for guided dental implant placement. The proposed system offers real‐time visualization, reduces reliance on static drilling guides, and eliminates the need for expensive specialized hardware, making it a cost‐effective and adaptable solution. In vitro testing showed consistent alignment between virtual overlays and physical models, successful guidance for implant drill positioning, and maintained clarity under proper lighting conditions.

However, challenges such as the system's reliance on stable lighting, visual cues, and operator dependence highlight areas for improvement. Future work will focus on quantitatively assessing accuracy, integrating AI for enhanced target recognition, and conducting clinical trials to validate the system in real‐world settings. Expanding its application to other dental procedures and exploring advanced hardware integrations may further optimize its performance.

In conclusion, the AR system presents a promising, eco‐friendly alternative for dental implantology, with the potential to improve accessibility, reduce costs, and enhance procedural flexibility.

## Author Contributions

Richard Mosch contributed to acquisition, analysis, and interpretation and drafted the manuscript. Vasilios Alevizakos contributed to conception and design and critically revised the manuscript. Dragan Alexander Ströbele contributed to analysis and drafted the manuscript. Marcus Schiller contributed to acquisition and critically revised the manuscript. Constantin von See contributed to conception and design and critically revised the manuscript. All authors gave their final approval and agreed to be accountable for all aspects of the work.

## Ethics Statement

The authors have nothing to report.

## Consent

The authors have nothing to report.

## Conflicts of Interest

The authors declare no conflicts of interest.

## Data Availability

The authors have nothing to report. The data sets used and/or analyzed during the current study are available from the corresponding author on reasonable request.
